# Supplementary material for: Identification of Daphnane Diterpenoids from Wikstroemia indica Using Liquid Chromatography with Tandem Mass Spectrometry
Source: Plants (Basel). 2023 Oct 19;12(20):3620. doi: 10.3390/plants12203620 (PMC10609749; doi:10.3390/plants12203620)
Supplement: Supplementary file 1 [file plants-12-03620-s001.zip › plants-2669211-supplementary.pdf]

Supporting Information

Identification of Daphnane Diterpenoids from *Wikstroemia indica* by Liquid Chromatography with Tandem Mass Spectrometry

Mi Zhang[1], Kouharu Otsuki[1,*], Reo Takahashi[1], Takashi Kikuchi[1], Di Zhou[2], Ning Li[2], and Wei Li[2,*]

[1]Faculty of Pharmaceutical Sciences, Toho University, Miyama 2-2-1, Funabashi, Chiba 274-8510, Japan

[2]School of Traditional Chinese Materia Medica, Key Laboratory of Innovative Traditional Chinese Medicine for Major Chronic Diseases of Liaoning Province, Key Laboratory for TCM Material Basis Study and Innovative Drug Development of Shenyang City, Shenyang Pharmaceutical University, Shenyang 110016, P. R. China

Contents

Spectroscopic data

Total ion chromatograms and extracted ion chromatograms in the positive ion mode from the E3-2 fraction, E3-3 fraction, and E3-4 fraction S1

^{1}H- and ^{13}C-NMR data of compounds **12**, **15**, **17**, **18**, **20**, and **21** S2

NMR spectra of compound **12** S6

HRESIMS data of compound **12** S10

NMR spectra of compound **15** S11

HRESIMS data of compound **15** S13

NMR spectra of compound **17** S14

HRESIMS data of compound **17** S16

NMR spectra of compound **18** S17

HRESIMS data of compound **18** S19

NMR spectra of compound **20** S20

HRESIMS data of compound **20** S22

NMR spectra of compound **21** S23

HRESIMS data of compound **21** S25

Product ion spectra of peaks **15** and **16** S26

Mass spectra and product ion spectra of peaks **3** and **21** S27

Product ion spectra of peaks **6**, **9**, **10**, **14**, **17**, and **19** S28

Product ion spectra of peaks **5**, **7**, 1**8**, and **20** S29

Product ion spectra of peaks **1**, **2**, **8**, and **11** S30

Product ion spectra of peaks **4** and **13** S31

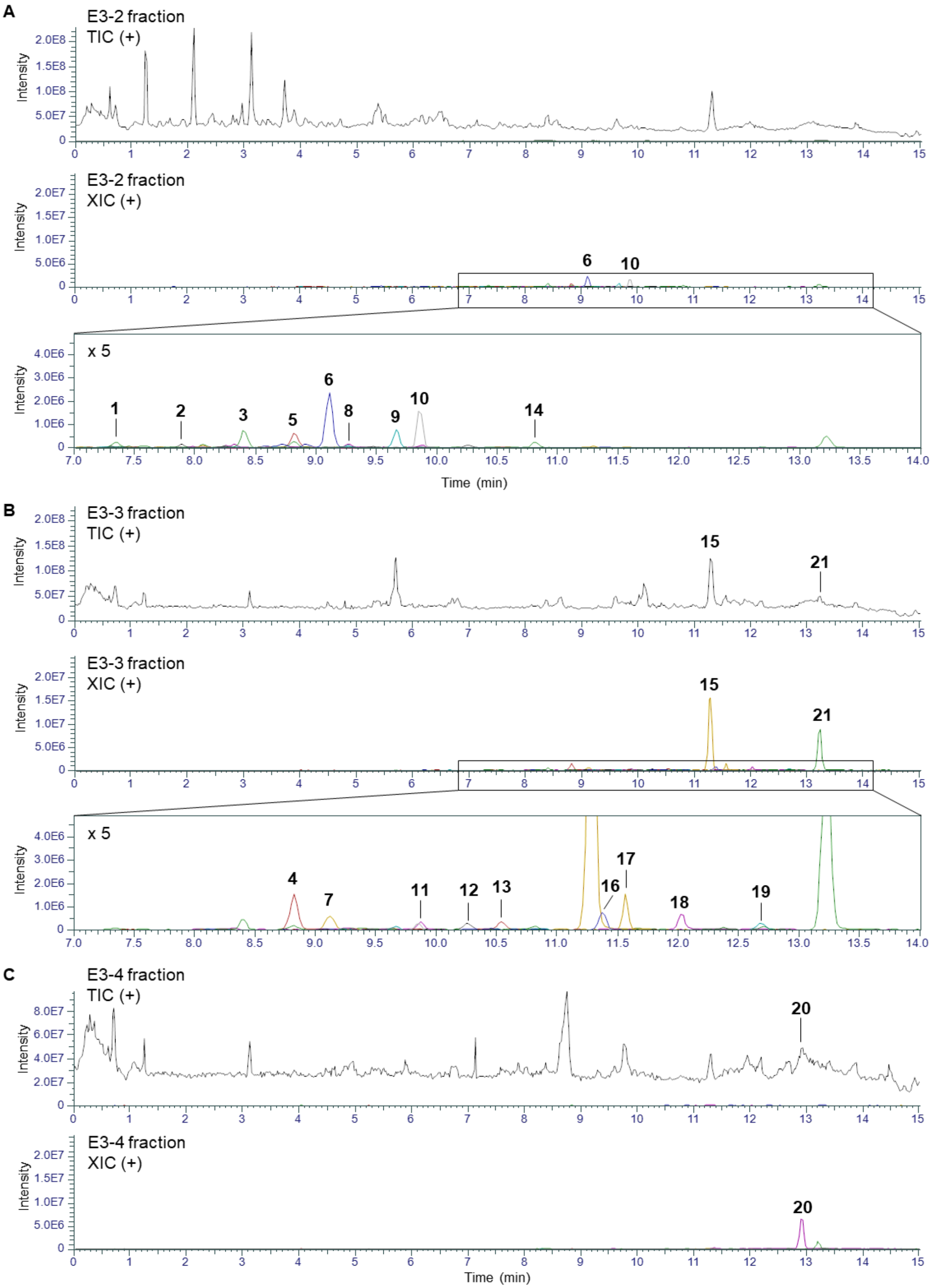

Figure S1. Total ion chromatograms and extracted ion chromatograms in the positive ion mode from the (A) E3-2 fraction, (B) E3-3 fraction, and (C) E3-4 fraction.

Table S1. ^1H (500 MHz) and ^{13}C (125 MHz) NMR data of compounds **15**, **21**, and **17** ($CDCl_3$).

	**15**		**17**		**21**	
no.	δ_H (J in Hz)	δ_C	δ_H (J in Hz)	δ_C	δ_H (J in Hz)	δ_C
1	7.59 (1H, dd, 2.5, 1.4)	161.4	7.56 (1H, m)	160.5	7.61 (1H, dd, 2.6, 1.4)	161.2
2		136.6		136.9		136.7
3		209.9		209.5		209.8
4		72.2		72.2		72.3
5	4.23 (1H, s)	72.1	4.23 (1H, s)	72.2	4.23 (1H, s)	72.0
6		60.4		60.4		60.4
7	3.41 (1H, s)	64.4	3.57 (1H, br s)	64.4	3.43 (1H, s)	64.2
8	2.88 (1H, d, 2.5)	36.7	3.57 (1H, d, 2.5)	35.6	2.92 (1H, d, 2.6)	36.7
9		78.7		78.2		79.5
10	3.72 (1H, dq, 2.8, 2.5)	48.2	3.81 (1H, q, 2.5)	47.5	3.79 (1H, dq, 2.9, 2.6)	48.2
11	2.43 (1H, dq, 8.6, 7.2)	34.8	2.47 (1H, q, 7.2)	44.2	2.46 (1H, dq, 8.6, 7.2)	34.9
12	1.62 (1H, d, 14.3)	36.5	5.09 (1H, br s)	78.4	1.66 (1H, d, 14.4)	36.5
	2.19 (1H, dd, 14.3, 8.6)				2.22 (1H, dd, 14.4, 8.6)	
13		84.1		83.8		84.4
14	4.34 (1H, d, 2.5)	81.8	4.80 (1H, d, 2.6)	80.5	4.40 (1H, d, 2.5)	82.0
15		146.3		143.0		146.1
16	4.87 (1H, t, 1.4)	111.1	4.99 (1H, br s)	113.6	4.89 (1H, t, 1.4)	111.3
	5.00 (1H, br s)		5.00 (1H, br s)		5.00 (1H, br s)	
17	1.76 (3H, s)	19.0	1.84 (1H, br s)	18.8	1.78 (3H, s)	18.9
18	1.14 (3H, d, 7.2)	20.3	1.33 (1H, d, 7.2)	18.2	1.17 (3H, d, 7.2)	20.4
19	1.78 (3H, dd, 2.8, 1.4)	9.9	1.77 (1H, m)	9.9	1.79 (3H, dd, 2.9, 1.4)	9.9
20	3.75 (1H, d, 12.6)	65.3	3.75 (1H, dd, 12.5, 4.5)	65.3	3.76 (1H, d, 12.5)	65.0
	3.86 (1H, d, 12.6)		3.95 (1H, dd, 12.5, 5.2)		3.87 (1H, d, 12.5)	
1'		119.4		117.1		116.5
2'	1.93 (2H, m)	34.9	5.65 (1H, d, 15.4)	122.4	5.69 (1H, d, 15.5)	122.8
3'	1.56 (2H, m)	23.5	6.66 (1H, dd, 15.4, 10.6)	135.1	6.68 (1H, dd, 15.5, 10.6)	138.9
4'	1.32 (2H, m)	29.6	6.04 (1H, dd, 15.1, 10.6)	128.6	6.04 (1H, dd, 15.2, 10.6)	134.7
5'	1.25 (2H, m)	29.6	5.85 (1H, dt. 15.1, 7.0)	139.3	5.83 (1H, d, 15.2, 7.2)	128.8
6'	1.25 (2H, m)	29.5	2.08 (2H, q, 7.0)	32.7	2.07 (2H, q, 7.2)	32.7
7'	1.25 (2H, m)	29.3	1.37 (2H, hex, 7.0)	28.7	1.24 (2H, m)	31.9
8'	1.23 (2H, m)	31.9	1.27 (2H, m)	31.3	1.24 (2H, m)	29.6
9'	1.25 (2H, m)	22.7	1.27 (2H, m)	22.5	1.24 (2H, m)	29.5
10'	0.86 (3H, t, 7.0)	14.1	0.87 (3H, t, 6.8)	14.0	1.24 (2H, m)	29.3
11'					1.36 (2H, m)	29.1
12'					1.36 (2H, m)	29.1
13'					1.28 (2H, m)	22.7

14'			0.86 (3H, t, 6.9)	14.1
1"		165.8		
2"	6.35 (1H, d, 15.9)	117.3		
3"	7.62 (1H, d, 15.9)	145.9		
4"		134.1		
5"	7.51 (2H, m)	128.9		
6"	7.37 (2H, m)	128.3		
7"	7.37 (2H, m)	130.6		
8"	7.37 (2H, m)	128.3		
9"	7.51 (2H, m)	128.9		

Table S2. ^{1}H (500 MHz) and ^{13}C (125 MHz) NMR data of compounds **12**, **18**, and **20** ($CDCl_3$).

	**12**		**18**		**20**	
no.	δ_H (*J* in Hz)	δ_C	δ_H (*J* in Hz)	δ_C	δ_H (*J* in Hz)	δ_C
1	2.97 (1H, t, 12.0)	49.4	2.11 (1H, brt, 9.2)	49.4	2.37 (1H, br t, 11.2)	50.7
2	1.84 (1H, m)	37.6	1.69 (1H, m)	36.9	1.87 (1H, m)	36.3
3	4.91 (1H, d, 4.9)	82.5	3.82 (1H)[a]	79.6	5.06 (1H, 4.9)	83.0
4		79.5		79.3		80.1
5	4.08 (1H, s)	73.7	3.81 (1H)[a]	71.6	4.09 (1H, br s)	73.5
6		60.7		60.0		61.0
7	3.37 (1H, s)	63.5	3.35 (1H, s)	64.4	3.32 (1H, s)	64.2
8	3.04 (1H, d, 2.9)	36.7	2.94 (1H, s)	36.6	2.87 (1H, d, 2.5)	36.8
9		81.4		80.9		81.2
10	3.03 (1H, d, 12.6)	48.2	2.95 (1H, d, 9.2)	48.0	3.08 (1H, d, 11.2)	47.3
11	2.82 (1H, t, 7.7)	41.2	2.49 (1H, dq, 8.3, 7.1)	35.4	2.64 (1H, dq, 8.0, 7.2)	35.3
12	1.96 (1H, m)<br>2.34 (1H, d, 14.7)	29.6	1.66 (1H, d, 14.1)<br>2.18 (1H, dd, 14.1, 8.3)	36.3	1.61 (1H, d, 14.0)<br>2.11 (1H, dd, 14.0, 8.0)	36.5
13		84.5		83.3		83.4
14	4.38 (1H, d, 2.9)	81.4	4.28 (1H, d, 2.6)	82.6	4.25 (1H, d, 2.5)	82.6
15		145.4		146.8		146.8
16	4.94 (1H, t, 1.3)<br>5.17 (1H, s)	111.9	4.86 (1H, t, 1.5)<br>4.98 (1H, brs)	110.8	4.83 (1H, t, 1.4)<br>4.94 (1H, br s)	110.8
17	1.81 (3H, s)	19.0	1.75 (3H, s)	18.9	1.72 (3H, s)	18.9
18	4.35 (1H, t, 10.5)<br>5.07 (1H, dd, 10.3, 2.0)	67.4	1.37 (3H, d, 7.1)	21.4	1.43 (3H, d, 7.2)	21.3
19	1.20 (3H, d, 6.9)	14.6	1.01 (3H, d, 6.9)	13.9	1.03 (3H, d, 6.6)	14.5
20	3.84 (2H, d, 7.2)	65.9	4.11 (1H, d, 11.7)<br>4.98 (1H, d, 11.7)	67.9	3.75 (1H, d, 12.2)<br>3.87 (1H, d, 12.2)	66.2
1'		118.5		119.8		119.7
2'	3.92 (1H, d, 7.5)	70.7	1.81, 1.97 (1H, m)	33.4	1.83, 1.99 (1H, m)	33.6
3'	1.48, 1.61 (1H, m)	28.6	1.60, 1.69 (1H, m)	21.0	1.62, 1.73 (1H, m)	21.0
4'	1.32, 1.67 (1H, m)	24.5	1.27, 1.33 (1H, m)	27.7	1.28, 1.38 (1H, m)	27.8
5'	1.45, 1.57 (1H, m)	19.3	1.27, 1.33 (1H, m)	27.8	1.28, 1.38 (1H, m)	28.0
6'	1.70, 1.88 (1H, m)	28.7	1.24, 1.39 (1H, m)	26.6	1.27, 1.45 (1H, m)	26.7
7'	5.36 (1H, dt, 11.2, 5.0)	73.4	1.15, 1.55 (1H, m)	24.8	1.17, 1.60 (1H, m)	24.7
8'	1.55, 2.00 (1H, m)	27.6	1.31 (2H, m)	37.6	1.39 (2H, m)	38.0
9'	2.39 (1H, m)	28.1	2.37 (1H, m)	29.4	2.54 (1H, m)	29.2
10'	1.26 (3H, d, 7.4)	18.7	0.79 (3H, d, 6.5)	13.0	0.82 (3H, d, 6.9)	12.6
3-Bz-CO		168.2				167.8
3-Bz-1		130.8				129.9
3-Bz-2,6	8.16 (2H, dd, 8.6, 1.5)	130.1			8.02 (2H, dd, 8.0, 1.1)	129.7
3-Bz-3,5	7.35 (2H, t, 7.5)	128.5			7.47 (2H, t, 8.0)	128.7
3-Bz-4	7.53 (1H, tt, 7.5, 1.3)	133.5			7.59 (1H, tt, 8.0, 1.1)	133.5
18-Bz-CO		166.0				
18-Bz-1		129.3				
18-Bz-2,6	8.05 (2H, dd, 8.5, 1.4)	129.6				
18-Bz-3,5	7.44 (2H, t, 7.5)	128.4				
18-Bz-4	7.54 (1H, tt, 7.5, 1.3)	132.8				

20-Bz-CO				166.7
20-Bz-1				129.9
20-Bz-2,6			8.06 (1H, dd, 8.0, 1.4)	129.9
20-Bz-3,5			7.44 (1H, t, 8.0)	128.5
20-Bz-4			7.55 (1H, tt, 8.0, 1.4)	133.2
7'-Bz-CO		166.5		
7'-Bz-1		129.3		
7'-Bz 2,6	8.11 (2H, dd, 8.3, 1.1)	129.6		
7'-Bz 3,5	7.47 (2H, t, 7.5)	128.3		
7'-Bz-4	7.58 (1H, tt, 7.5, 1.3)	133.0		

[a]Overlapping resonances.

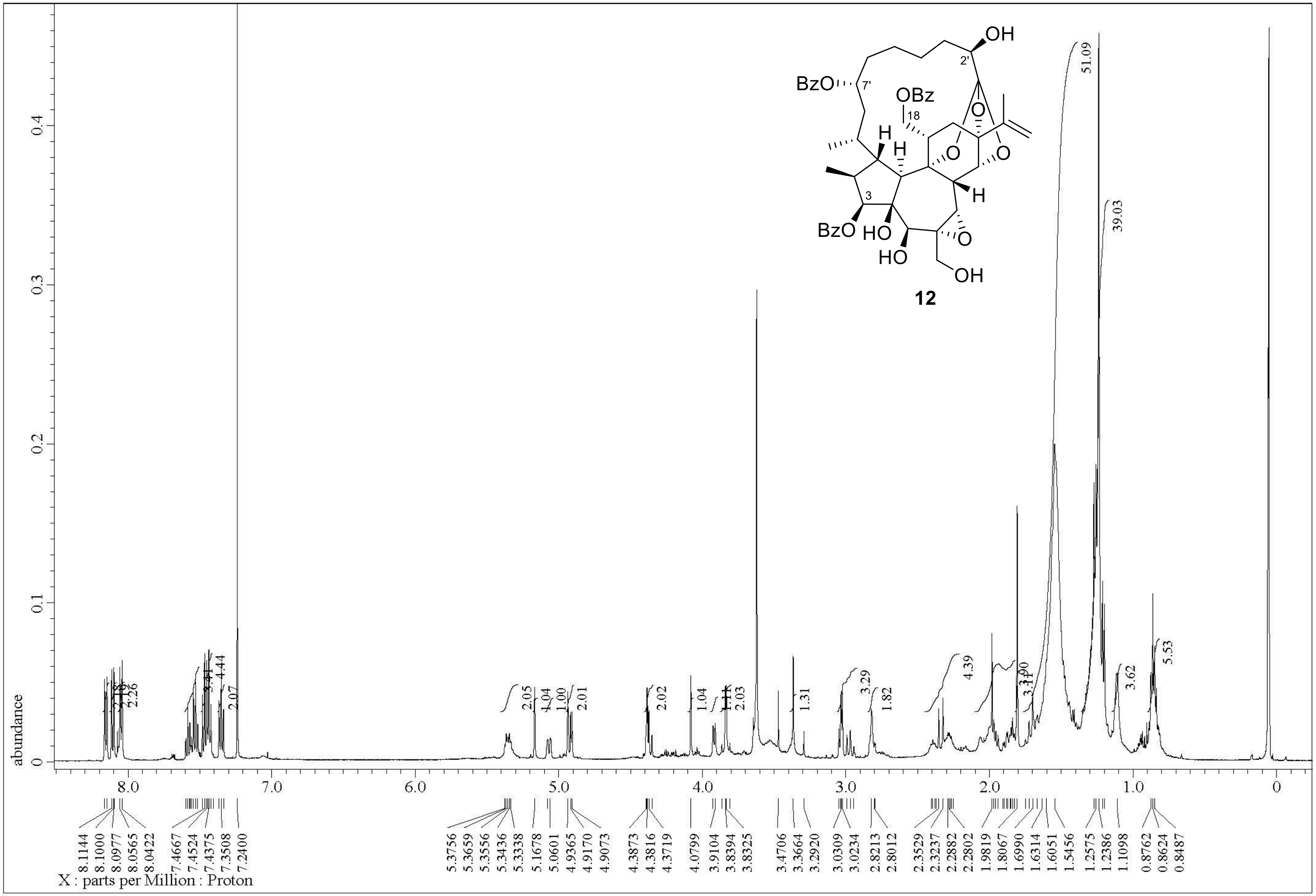

Figure S2. ^{1}H-NMR spectrum of compound **12**.

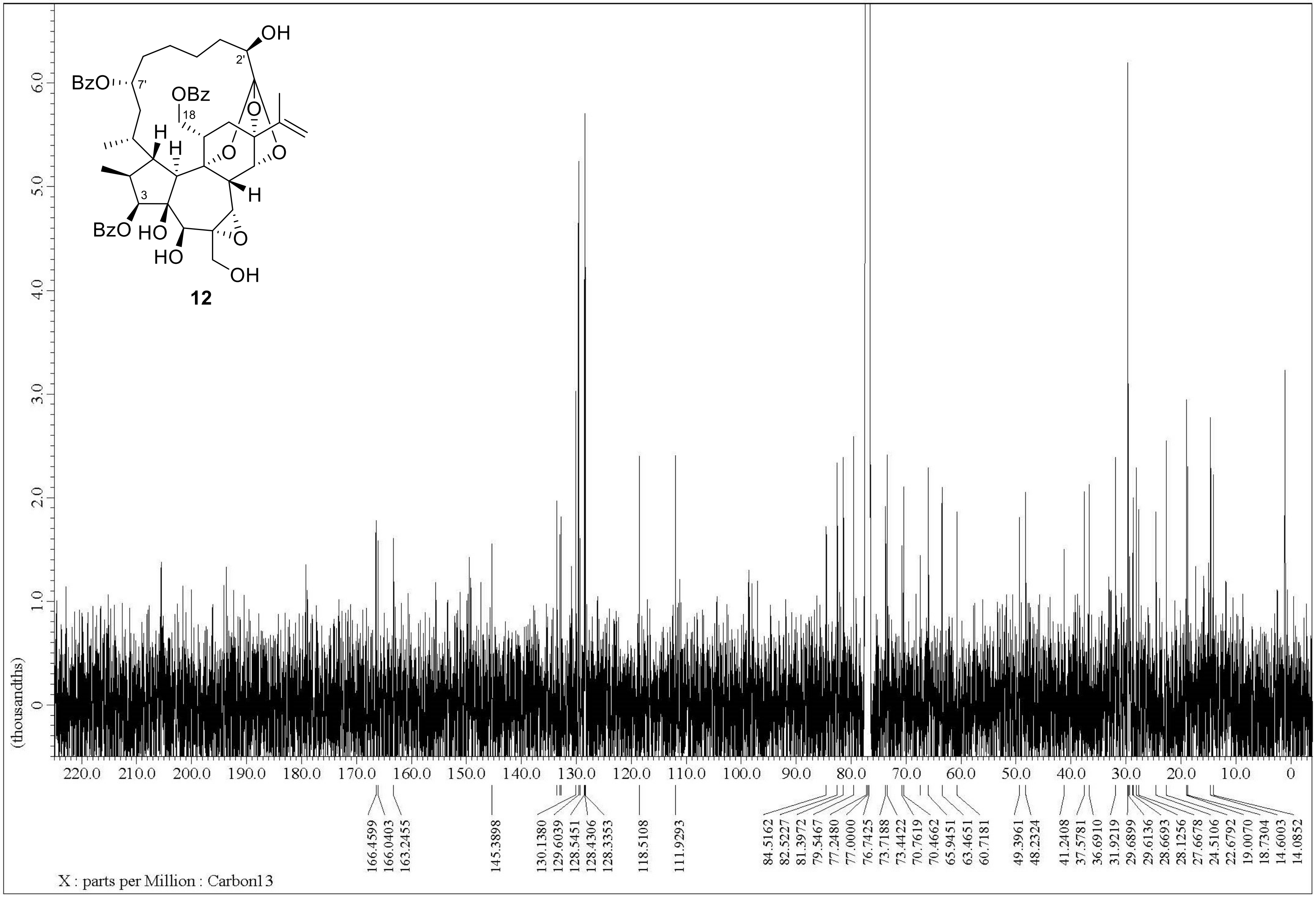

Figure S3. ^{13}C-NMR spectrum of compound **12**.

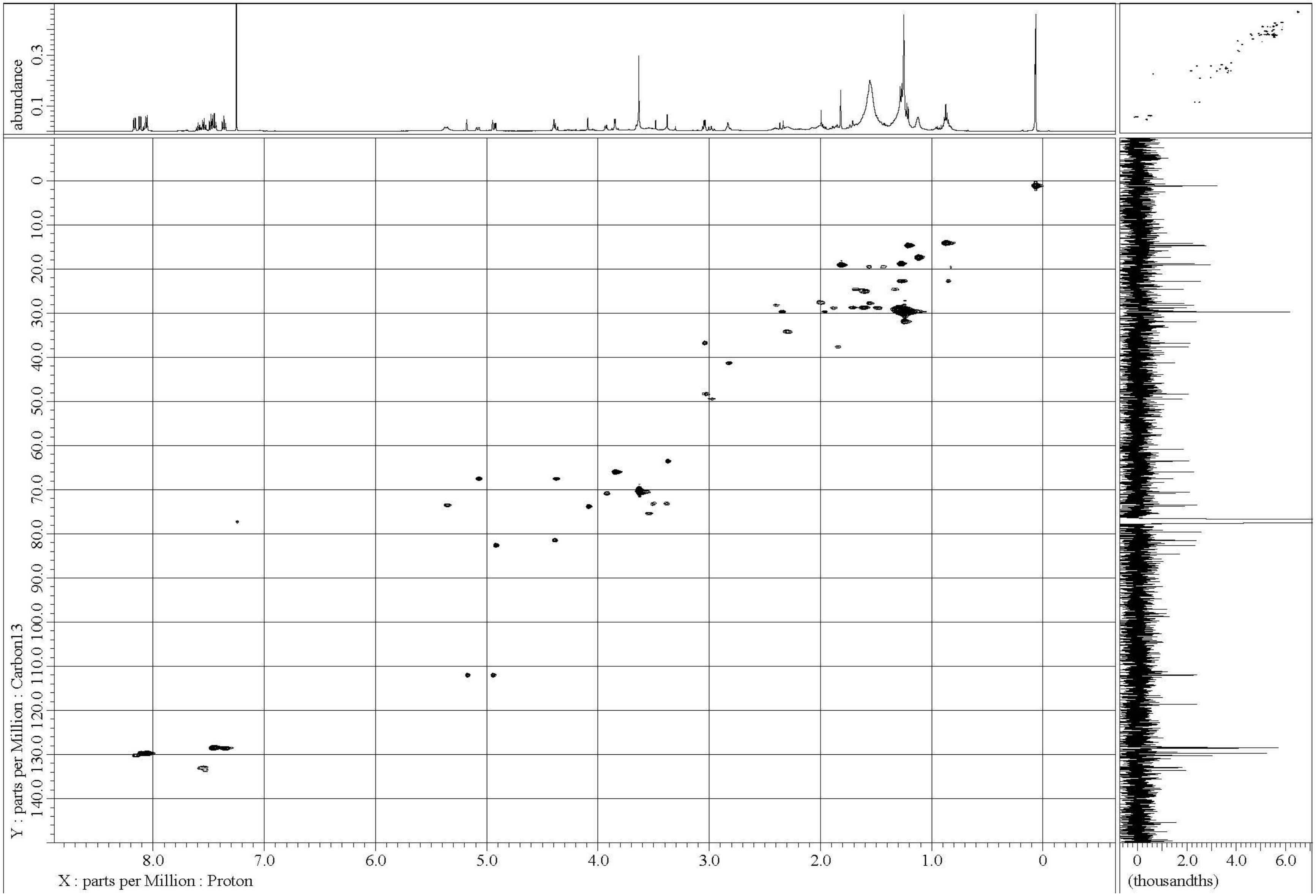

Figure S4. HSQC spectrum of compound **12**.

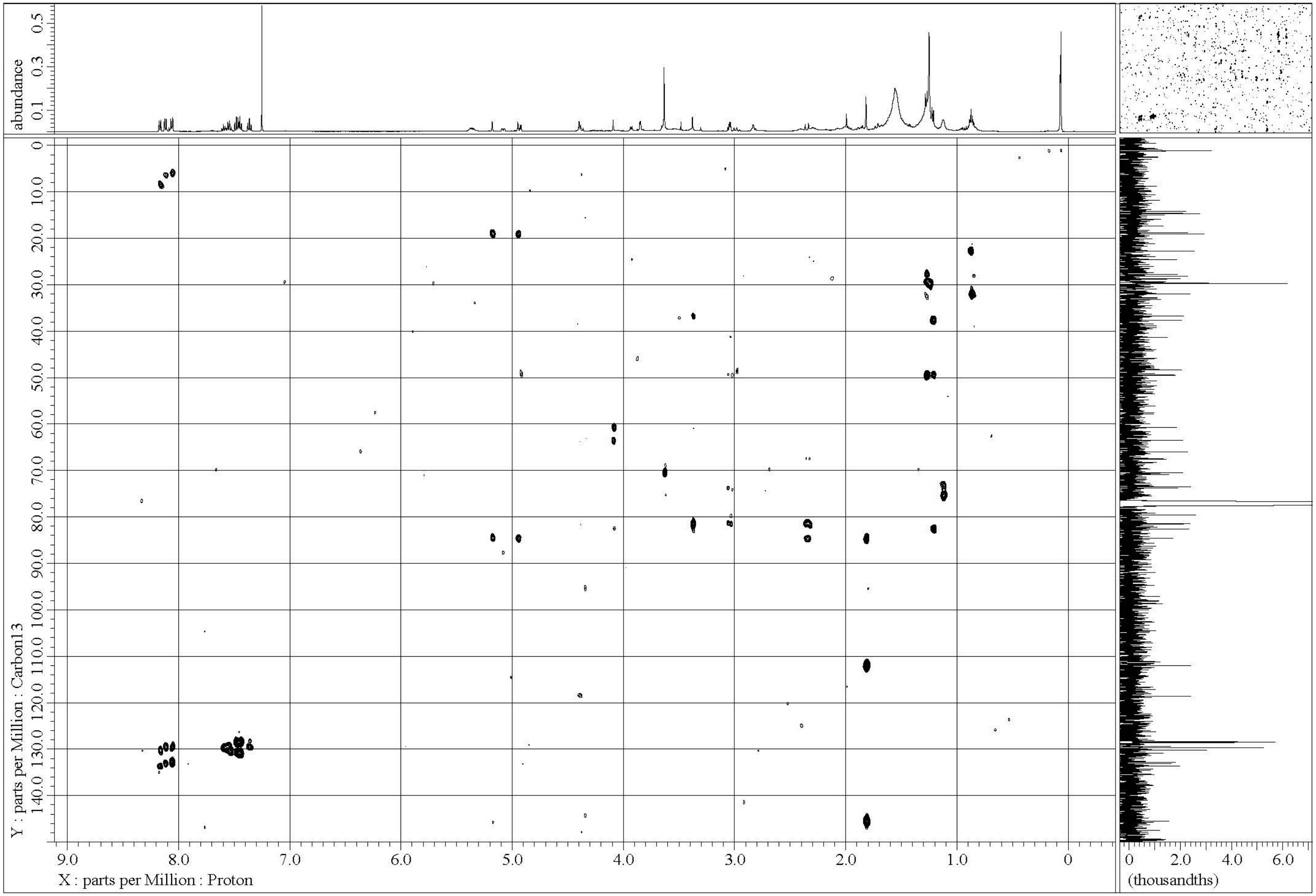

Figure S5. HMBC spectrum of compound **12**.

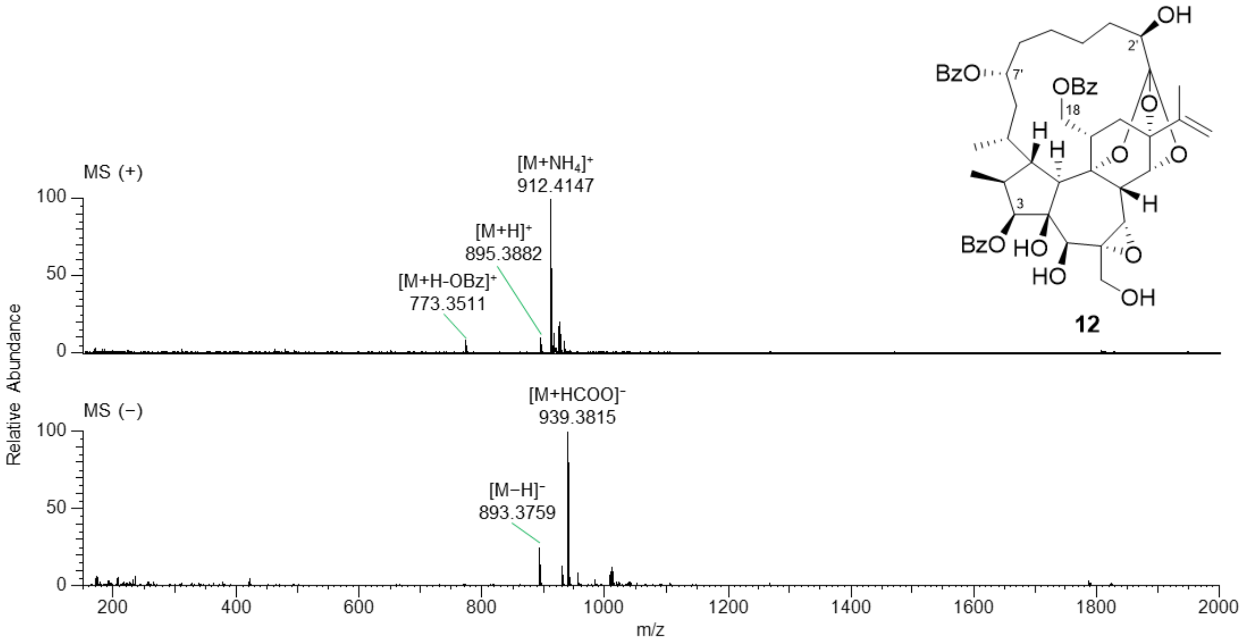

Peak Mass	Display Formula	Combined Fit	RDB	Delta [ppm]	Theo. mass	Rank	Combined Score	# Matched Iso.	# Missed Iso.	MS Cov. [%]	Pattern Cov. [%]
912.4147	$C_{51}H_{62}O_{14}N$	49.436437522999621.50		-1.98	912.41648	1	90.91	5	1	93.21	99.93
Fragment Mass											

Peak Mass	Display Formula	Combined Fit	RDB	Delta [ppm]	Theo. mass	Rank	Combined Score	# Matched Iso.	# Missed Iso.	MS Cov. [%]	Pattern Cov. [%]
939.3815	$C_{52}H_{59}O_{16}$	53.451450568803423.50		0.65	939.38086	1	97.55	5	1	100	99.91
Fragment Mass											

Figure S6. HRESIMS data of isolated compound **12** in positive ion mode (upper) and negative ion mode (lower).

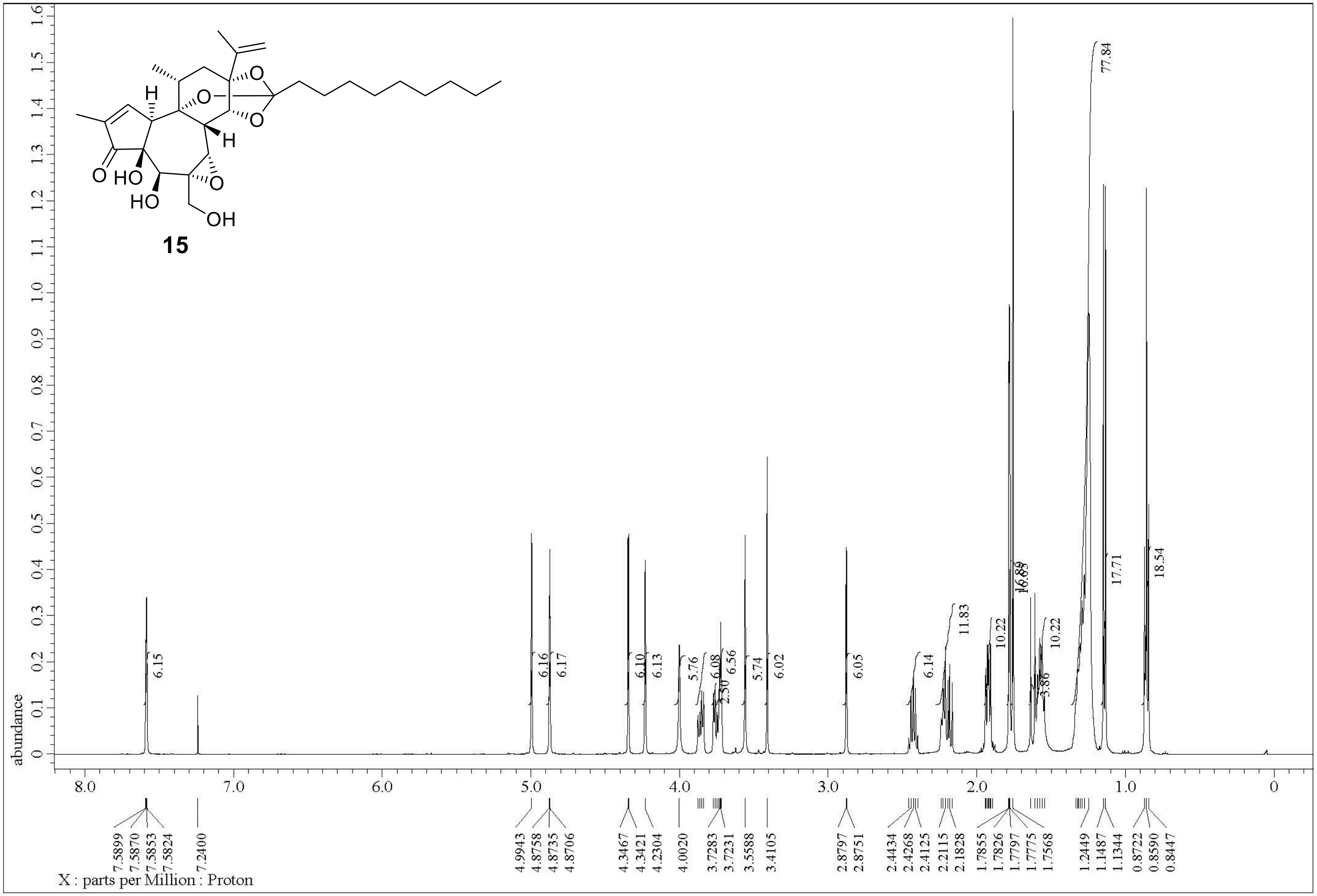

Figure S7. ^{1}H-NMR spectrum of compound **15**.

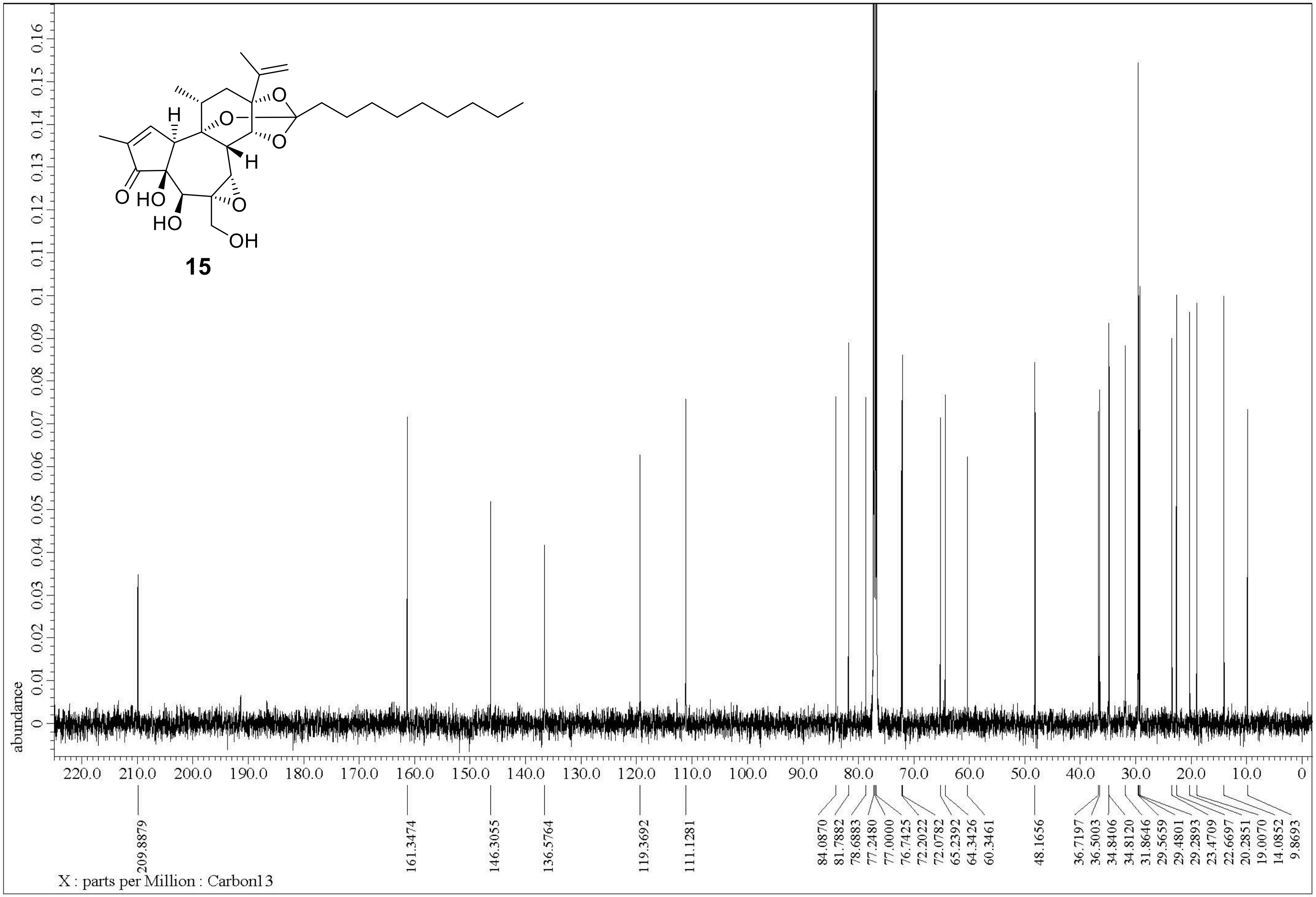

Figure S8. ^{13}C-NMR spectrum of compound **15**.

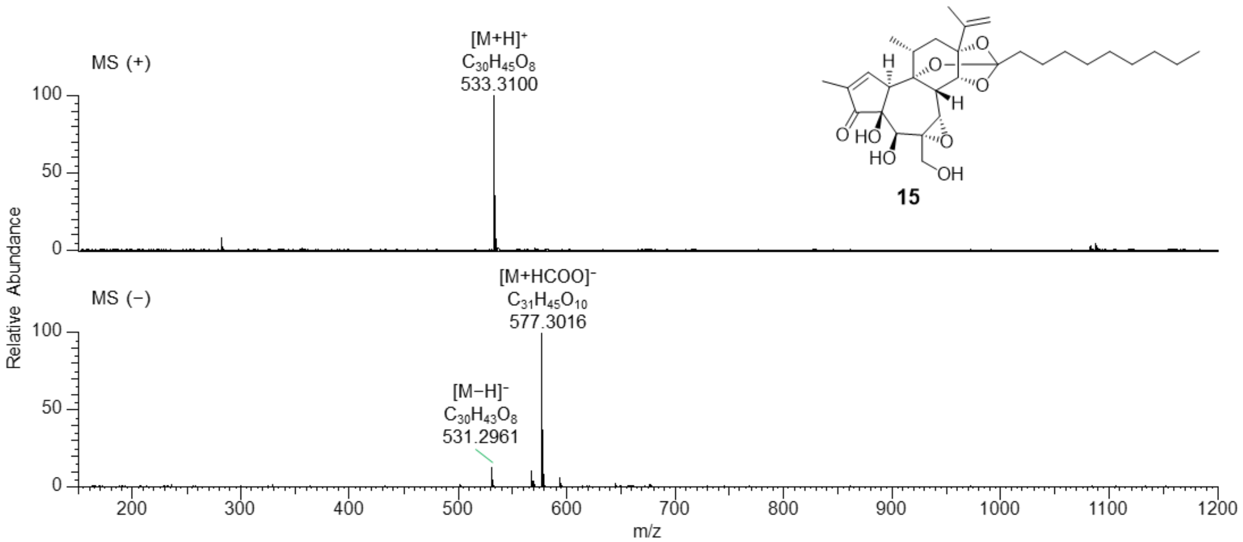

Peak Mass	Display Formula	Combined Fit	RDB	Delta [ppm]	Theo. mass	Rank	Combined Score	# Matched Iso.	# Missed Iso.	MS Cov. [%]	Pattern Cov. [%]
533.3100	$C_{30}H_{45}O_8$	46.29006226027728.50		-1.68	533.31089	1	97.03	5	0	99.85	99.91
Fragment Mass											

Peak Mass	Display Formula	Combined Fit	RDB	Delta [ppm]	Theo. mass	Rank	Combined Score	# Matched Iso.	# Missed Iso.	MS Cov. [%]	Pattern Cov. [%]
577.3018	$C_{31}H_{45}O_{10}$	83.85757239995389.50		0.00	577.30182	1	99.15	5	0	100	100
Fragment Mass											

Figure S9. HRESIMS data of isolated compound **15** in positive ion mode (upper) and negative ion mode (lower).

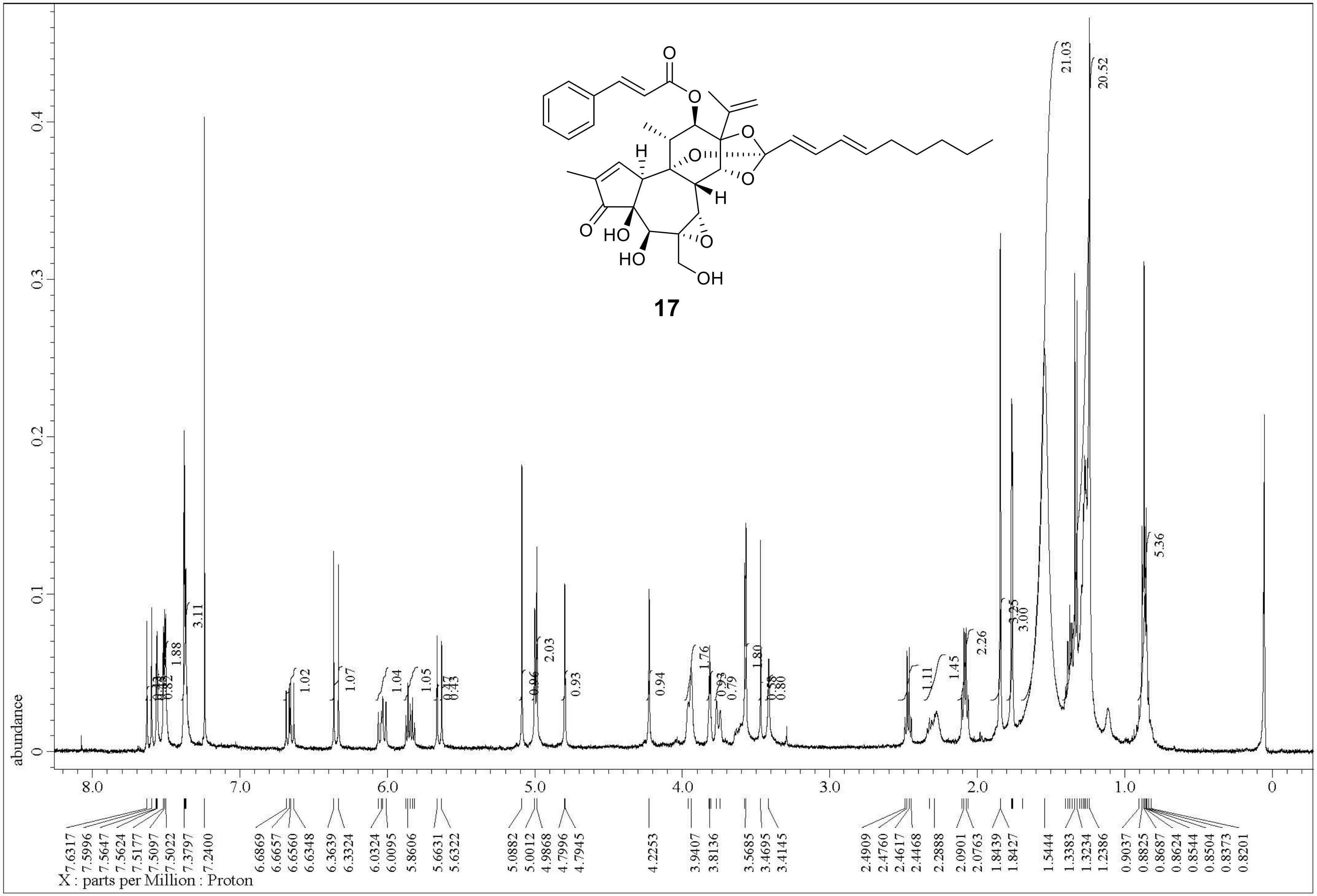

Figure S10. ^{1}H-NMR spectrum of compound **17**.

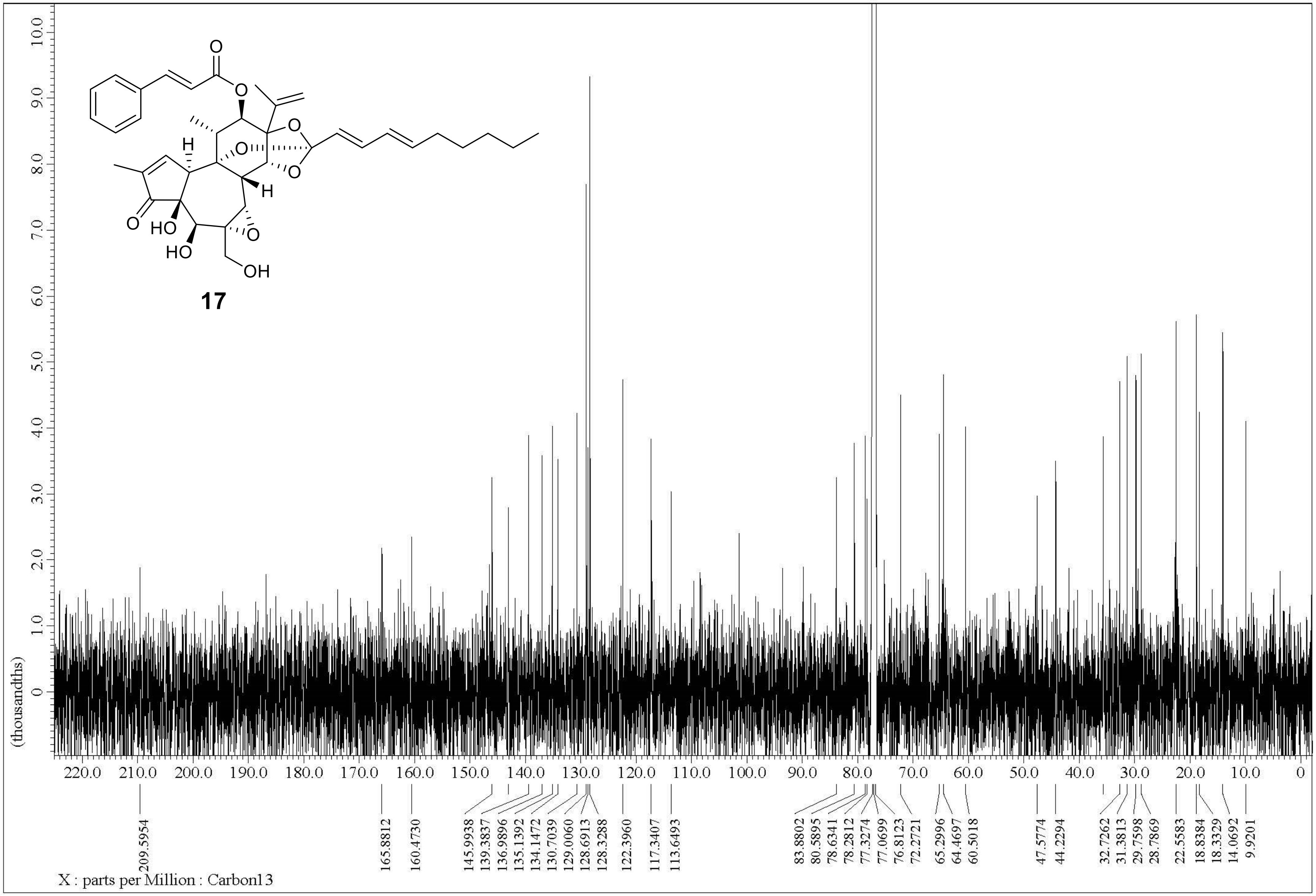

Figure S11. ^{13}C-NMR spectrum of compound **17**.

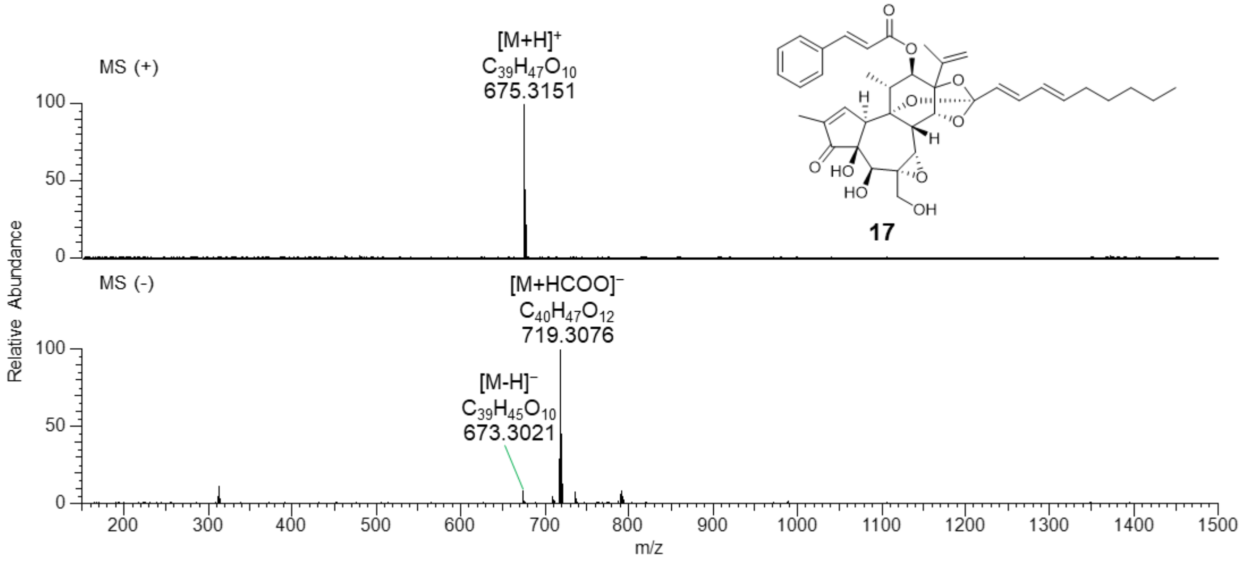

Peak Mass	Display Formula	Combined Fit	RDB	Delta [ppm]	Theo. mass	Rank	Combined Score	# Matched Iso.	# Missed Iso.	MS Cov. [%]	Pattern Cov. [%]
675.3151	$C_{39}H_{47}O_{10}$	65.9961893811134	16.50	-1.85	675.31637	1	98.21	5	0	100	100
Fragment Mass											

Peak Mass	Display Formula	Combined Fit	RDB	Delta [ppm]	Theo. mass	Rank	Combined Score	# Matched Iso.	# Missed Iso.	MS Cov. [%]	Pattern Cov. [%]
719.3076	$C_{40}H_{47}O_{12}$	61.3987308490693	17.50	1.97	719.30620	1	97.88	5	0	99.9	100
Fragment Mass											

Figure S12. HRESIMS data of isolated compound **17** in positive ion mode (upper) and negative ion mode (lower).

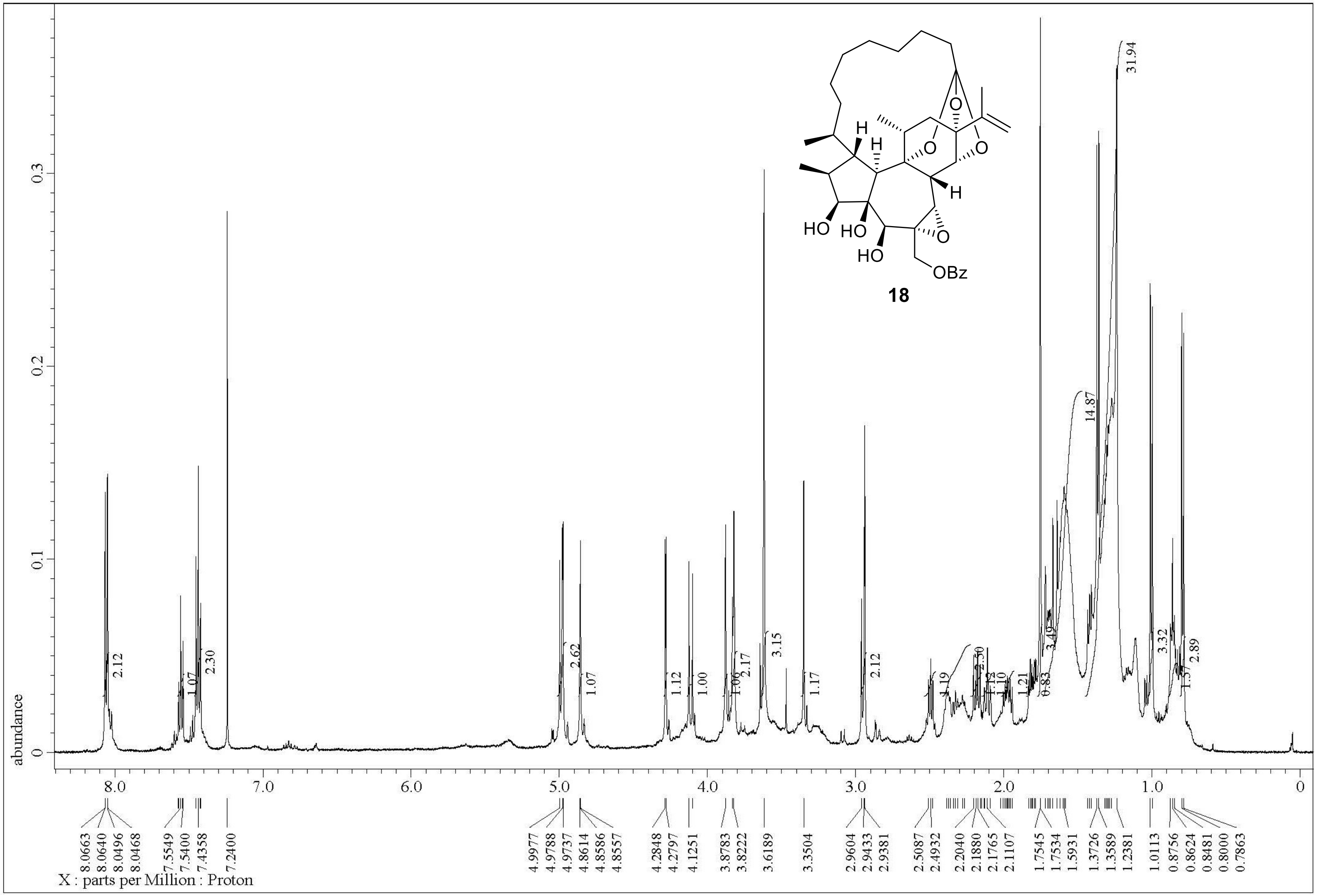

Figure S13. ^{1}H-NMR spectrum of compound **18**.

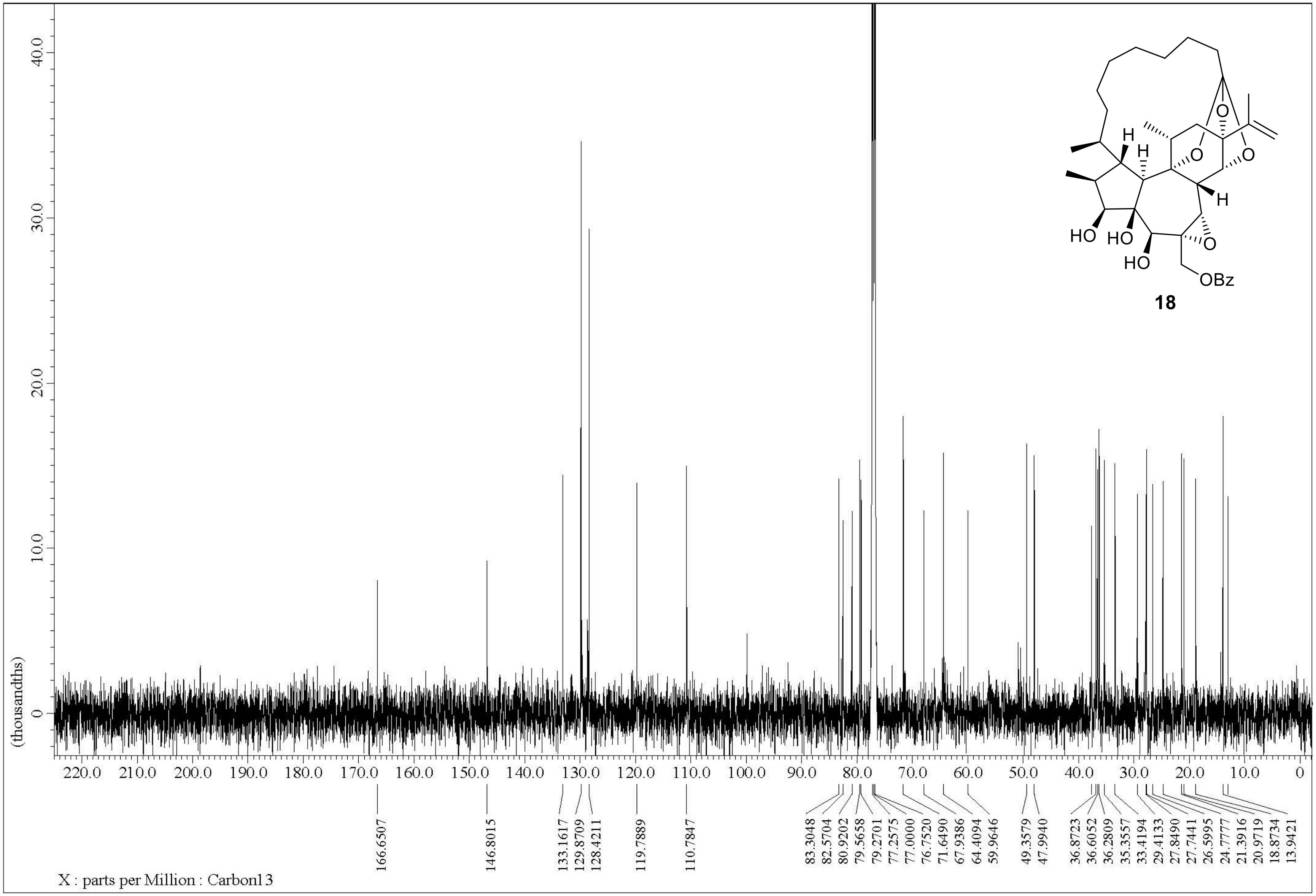

Figure S14. ^{13}C-NMR spectrum of compound **18**.

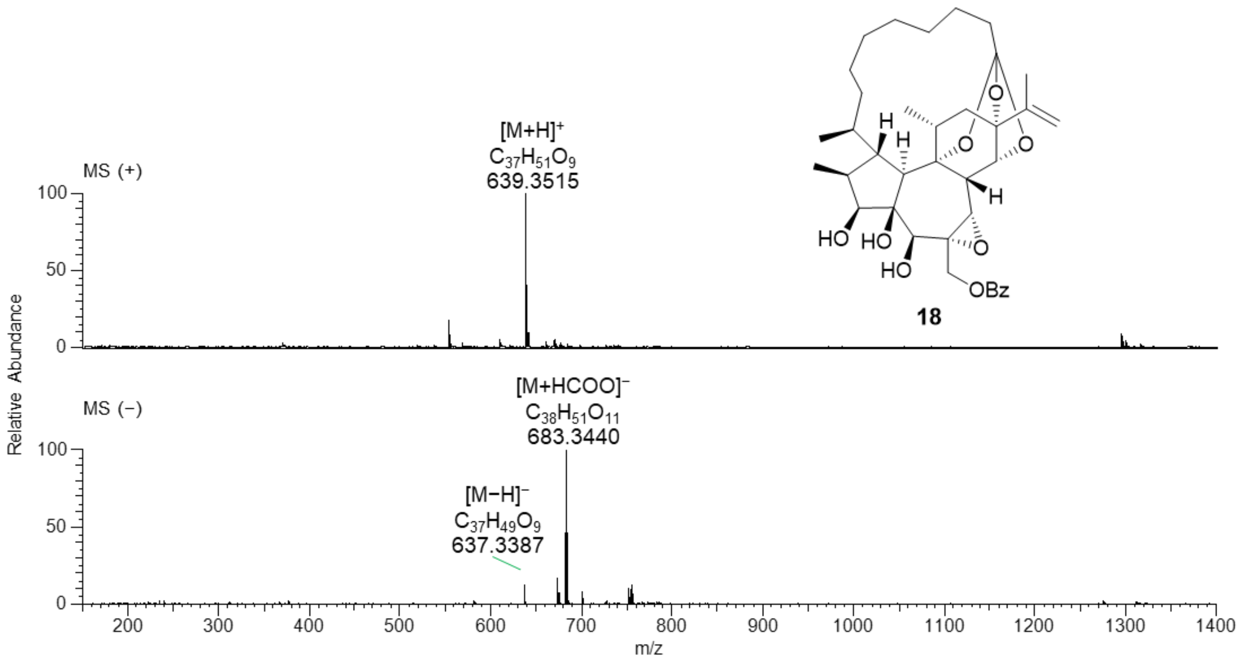

Peak Mass	Display Formula	Combined Fit	RDB	Delta [ppm]	Theo. mass	Rank	Combined Score	# Matched Iso.	# Missed Iso.	MS Cov. [%]	Pattern Cov. [%]
639.3515	$C_{37}H_{51}O_9$	67.9969457257302	12.50	-1.97	639.35276	1	98.32	5	0	100	100
Fragment Mass											

Peak Mass	Display Formula	Combined Fit	RDB	Delta [ppm]	Theo. mass	Rank	Combined Score	# Matched Iso.	# Missed Iso.	MS Cov. [%]	Pattern Cov. [%]
683.3440	$C_{38}H_{51}O_{11}$	67.1728422080105	13.50	0.45	683.34369	1	98.27	5	0	100	100
Fragment Mass											

Figure S15. HRESIMS data of compound **18** in positive ion mode (upper) and negative ion mode (lower).

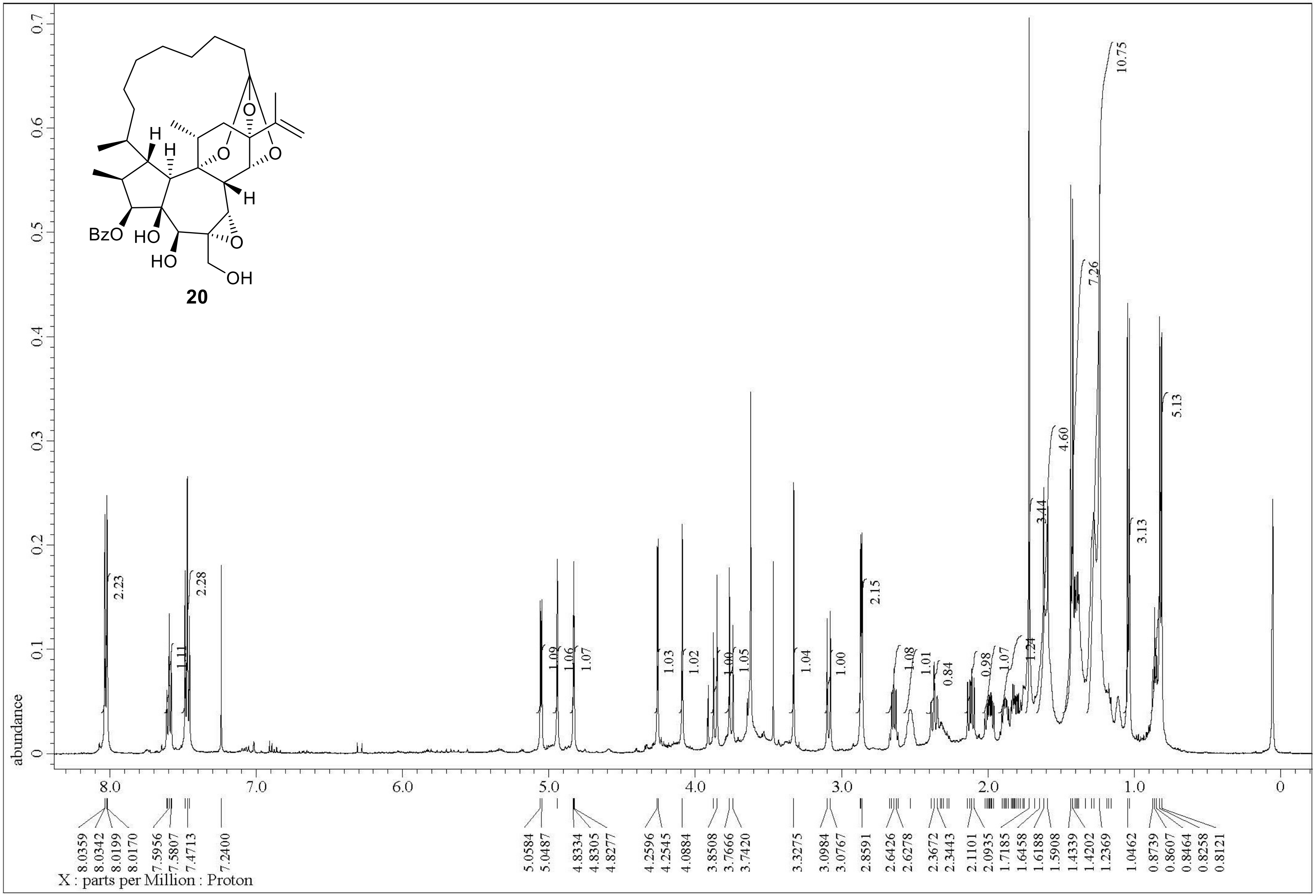

Figure S16. ^{1}H-NMR spectrum of compound **20**.

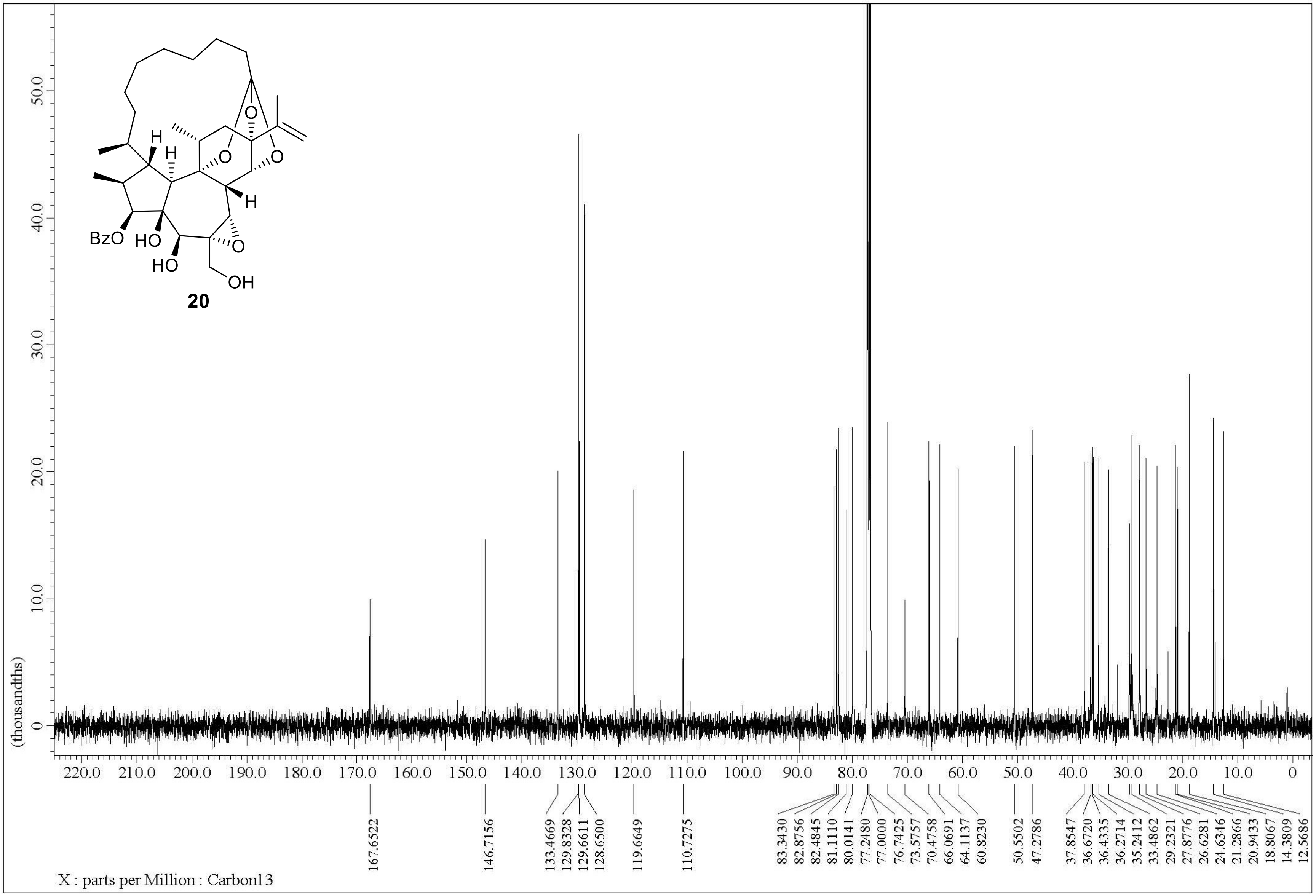

Figure S17. ^{13}C-NMR spectrum of compound **20**.

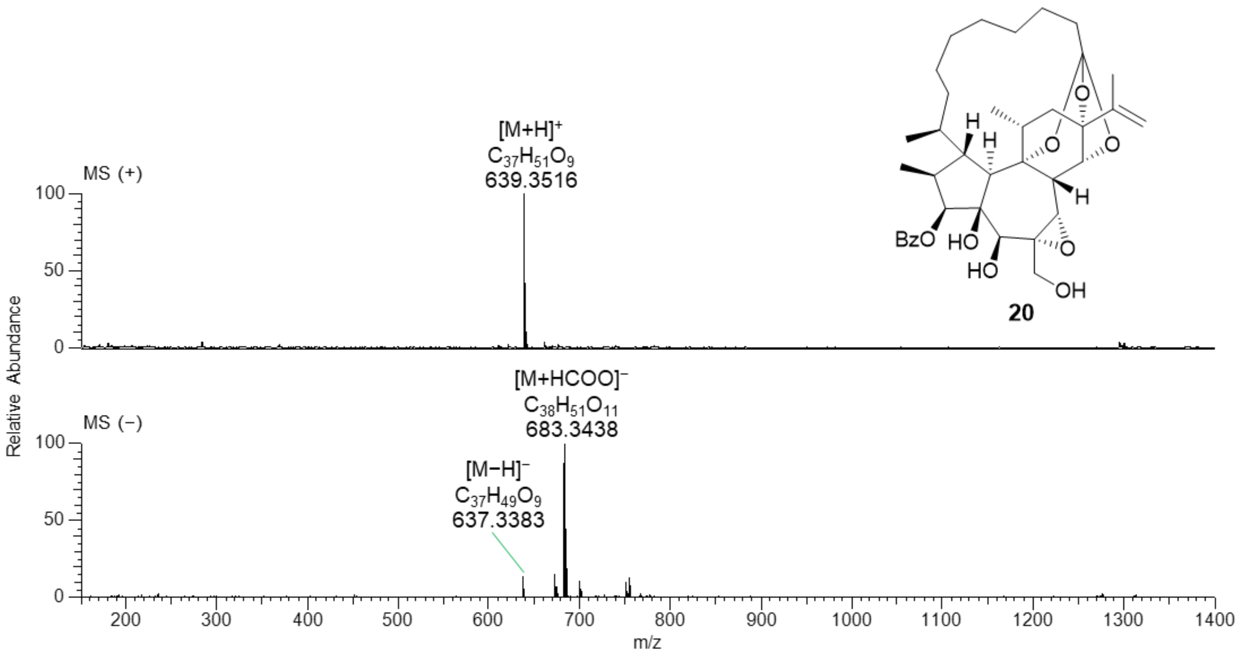

Peak Mass	Display Formula	Combined Fit	RDB	Delta [ppm]	Theo. mass	Rank	Combined Score	# Matched Iso.	# Missed Iso.	MS Cov. [%]	Pattern Cov. [%]
639.3516	$C_{37}H_{51}O_9$	47.1455941276345	12.50	-1.87	639.35276	1	97.08	4	1	99.85	99.84
Fragment Mass											

Peak Mass	Display Formula	Combined Fit	RDB	Delta [ppm]	Theo. mass	Rank	Combined Score	# Matched Iso.	# Missed Iso.	MS Cov. [%]	Pattern Cov. [%]
683.3438	$C_{38}H_{51}O_{11}$	80.3687003106102	13.50	0.09	683.34369	1	98.97	5	0	100	100
Fragment Mass											

Figure S18. HRESIMS data of isolated compound **20** in positive ion mode (upper) and negative ion mode (lower).

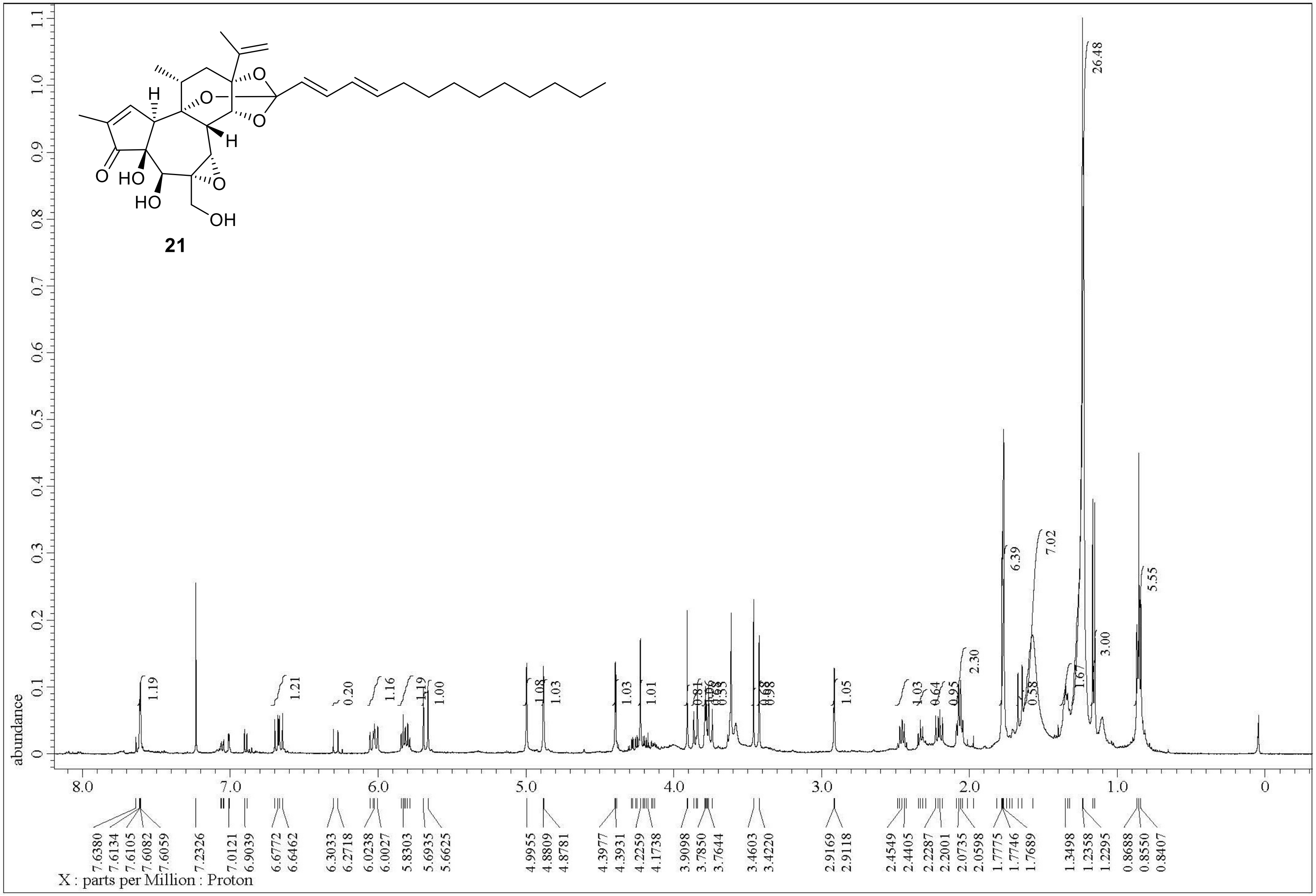

Figure S19. ^{1}H-NMR spectrum of compound **21**.

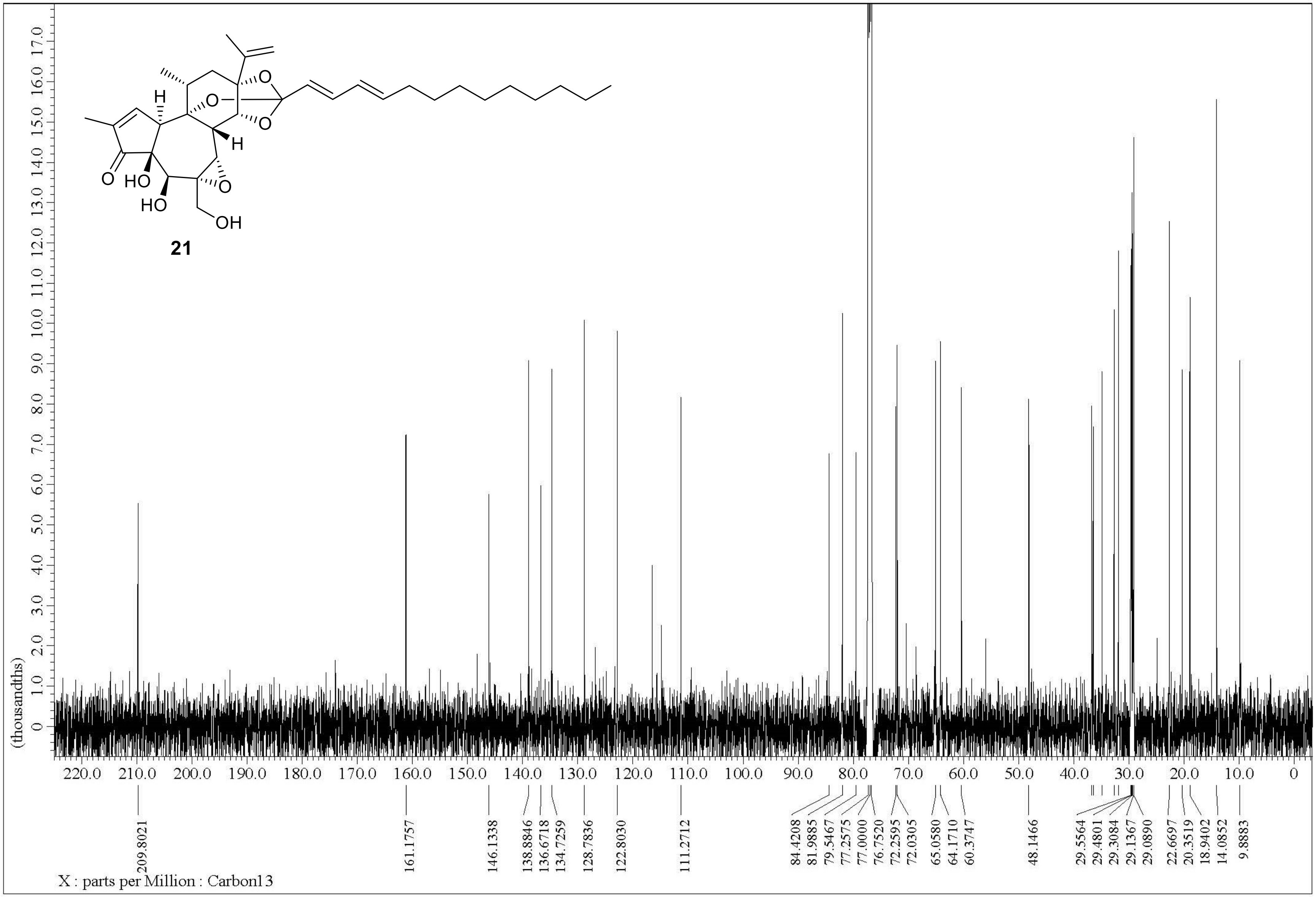

Figure S20. ^{13}C-NMR spectrum of compound **21**.

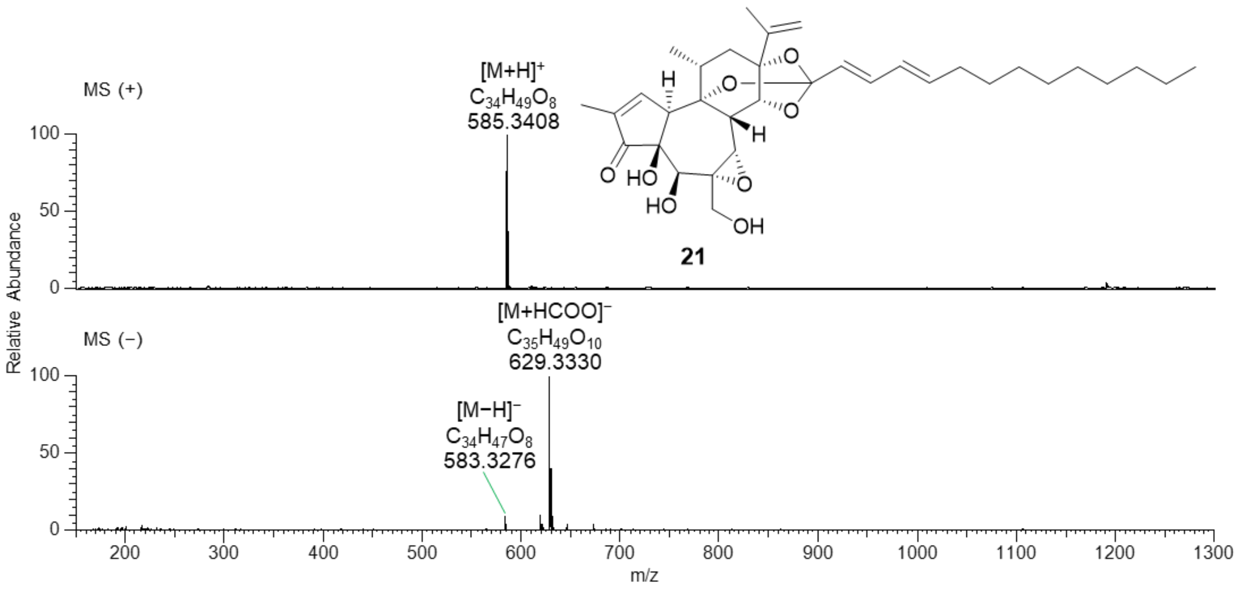

Peak Mass	Display Formula	Combined Fit	RDB	Delta [ppm]	Theo. mass	Rank	Combined Score	# Matched Iso.	# Missed Iso.	MS Cov. [%]	Pattern Cov. [%]
585.3408	$C_{34}H_{49}O_8$	61.2725506191585	10.50	-2.35	585.34219	1	97.93	5	0	99.96	100
Fragment Mass											

Peak Mass	Display Formula	Combined Fit	RDB	Delta [ppm]	Theo. mass	Rank	Combined Score	# Matched Iso.	# Missed Iso.	MS Cov. [%]	Pattern Cov. [%]
629.3330	$C_{35}H_{49}O_{10}$	83.4164824568874	11.50	-0.18	629.33312	1	98.97	5	0	99.83	100
Fragment Mass											

Figure S21. HRESIMS data of compound **21** in positive ion mode (upper) and negative ion mode (lower).

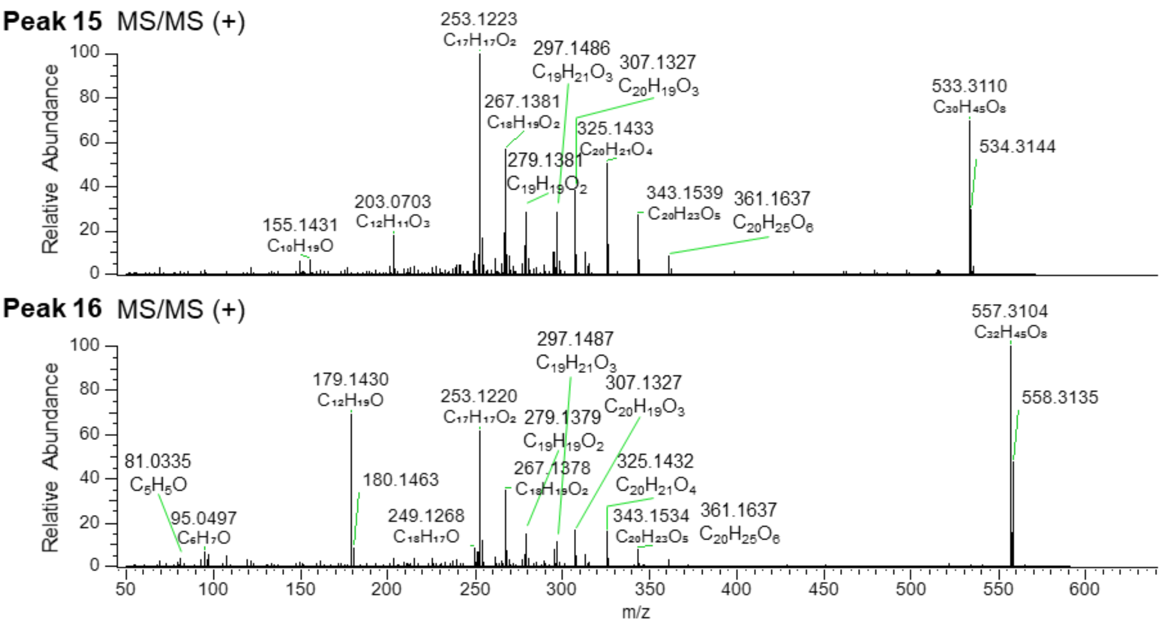

Figure S22. Product ion spectra of peaks **15** and **16** obtained from the protonated molecular ion peak in positive ion mode.

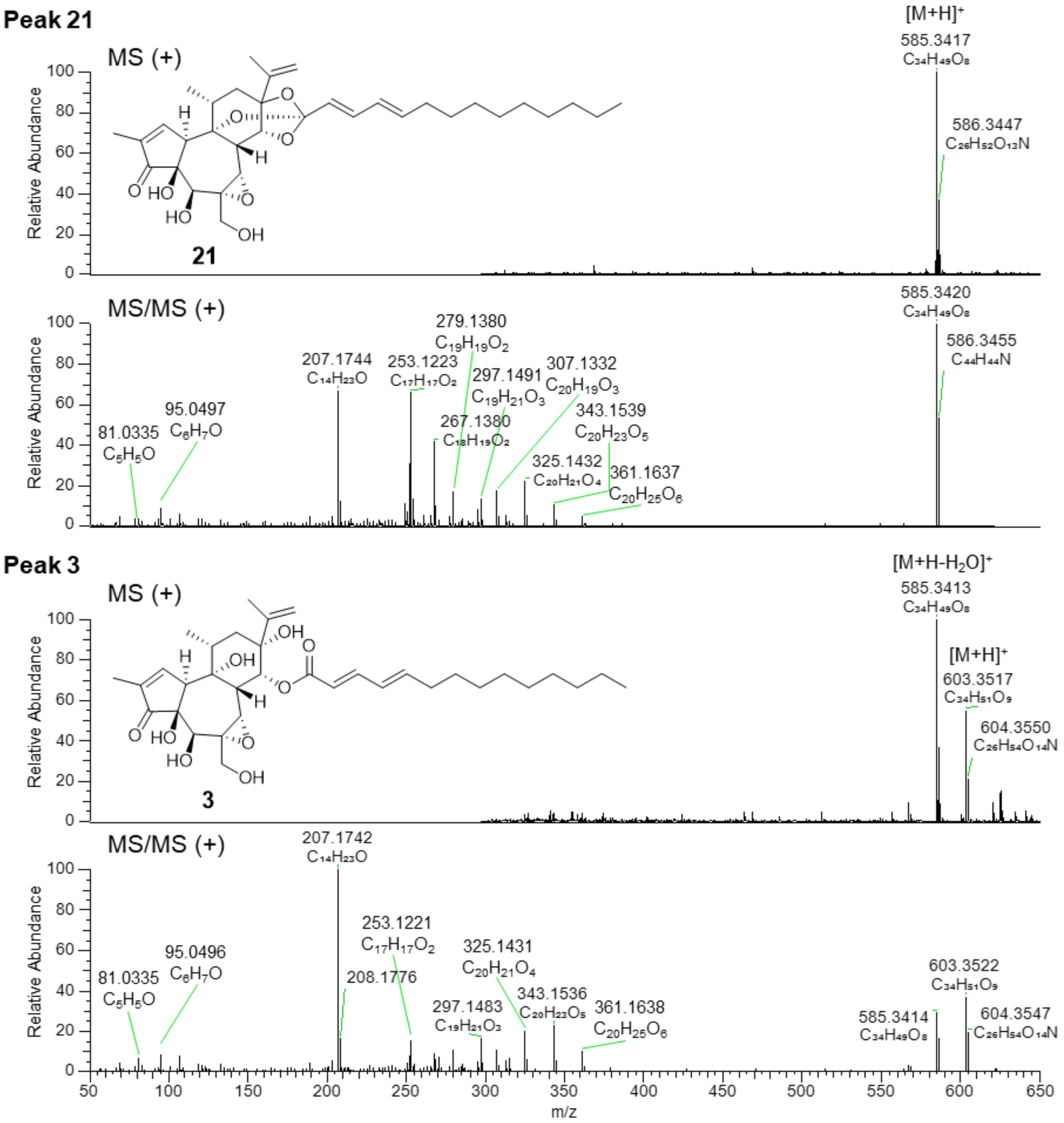

Figure S23. Mass spectra and product ion spectra of peaks **21** and **3** obtained from the protonated molecular ion peak in positive ion mode.

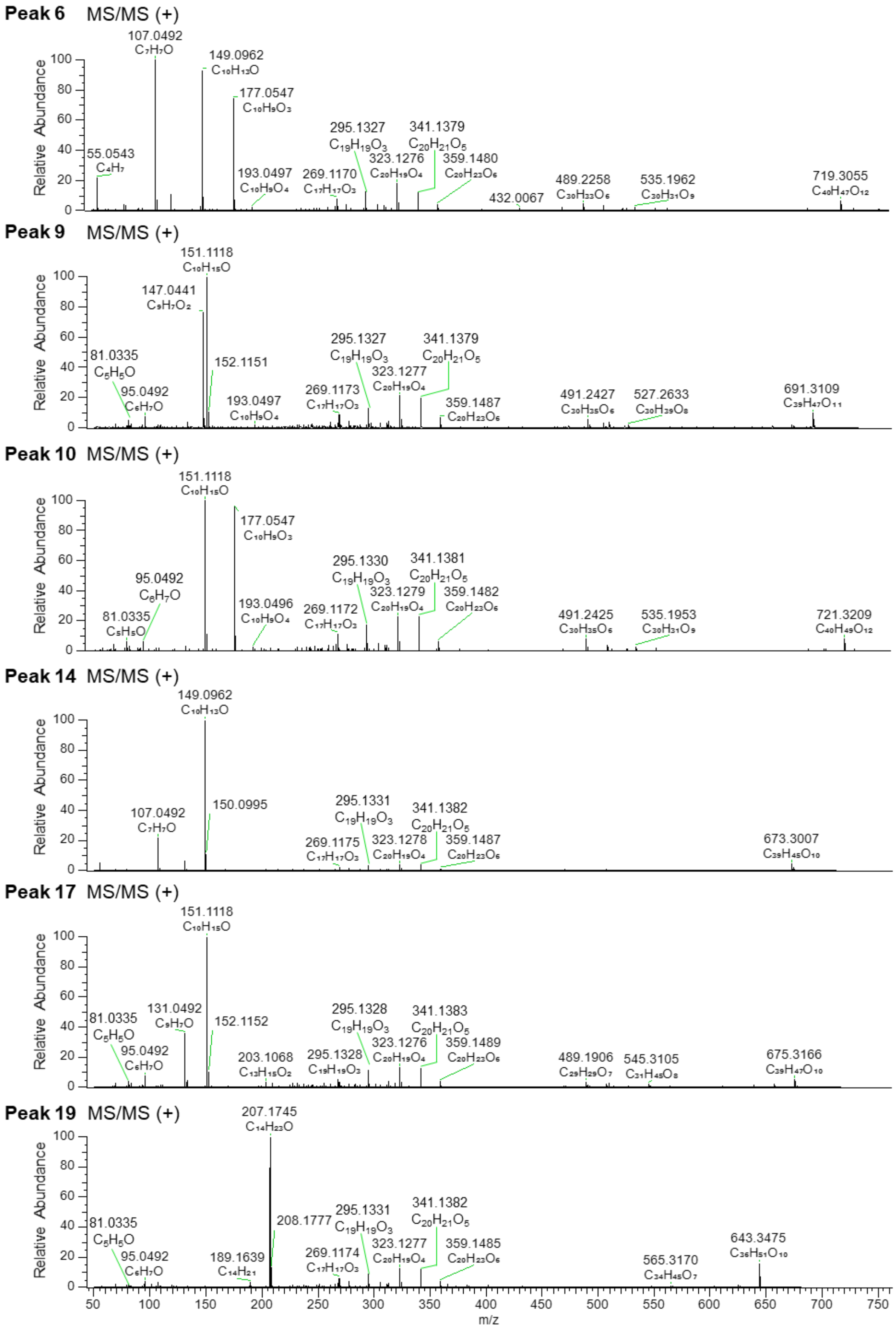

Figure S24. Product ion spectra of peaks **6**, **9**, **10**, **14**, **17**, and **19** obtained from the protonated molecular ion peak in positive ion mode.

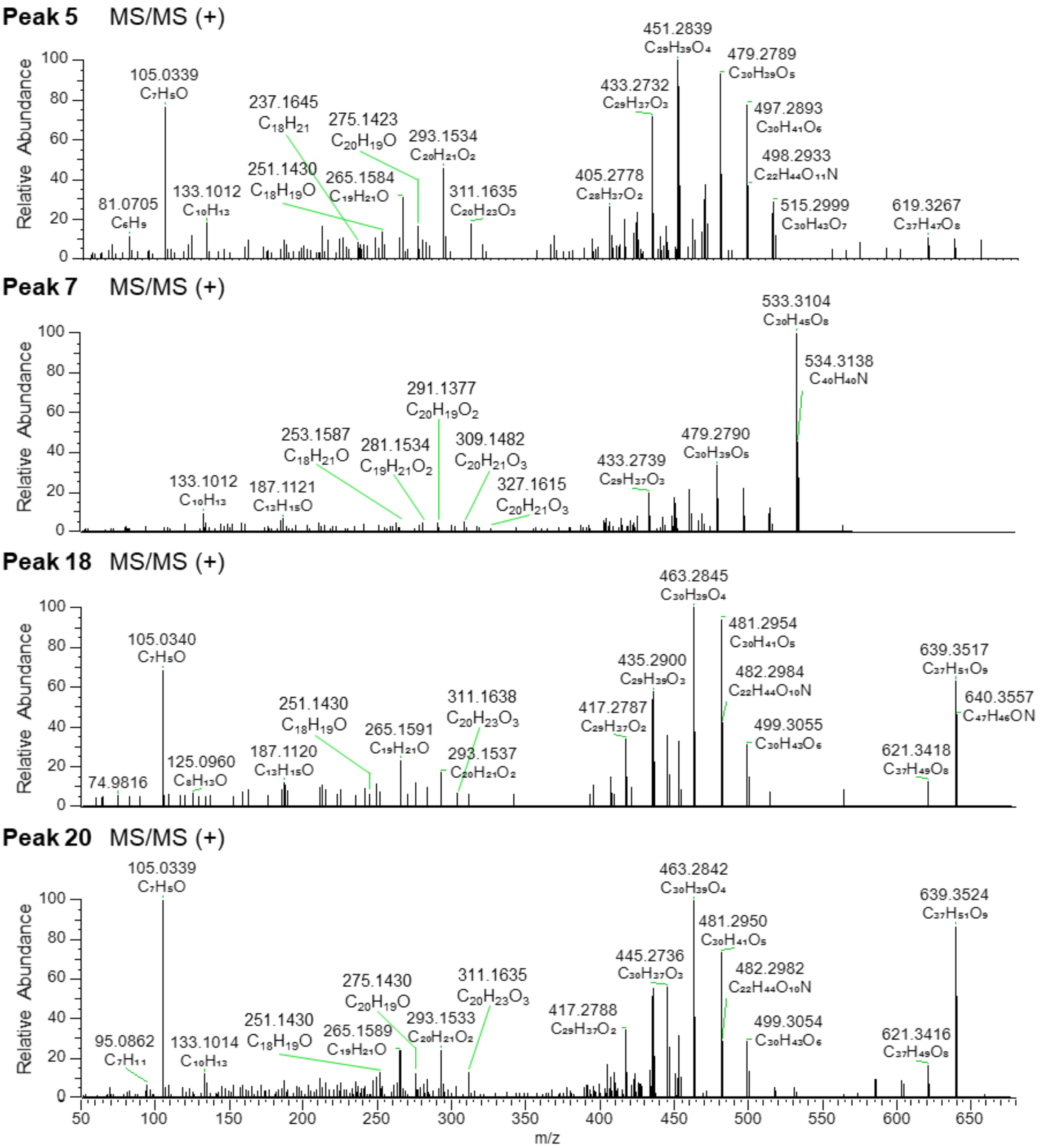

Figure S25. Product ion spectra of peaks **5**, **7**, **18**, and **20** obtained from the protonated molecular ion peak in positive ion mode.

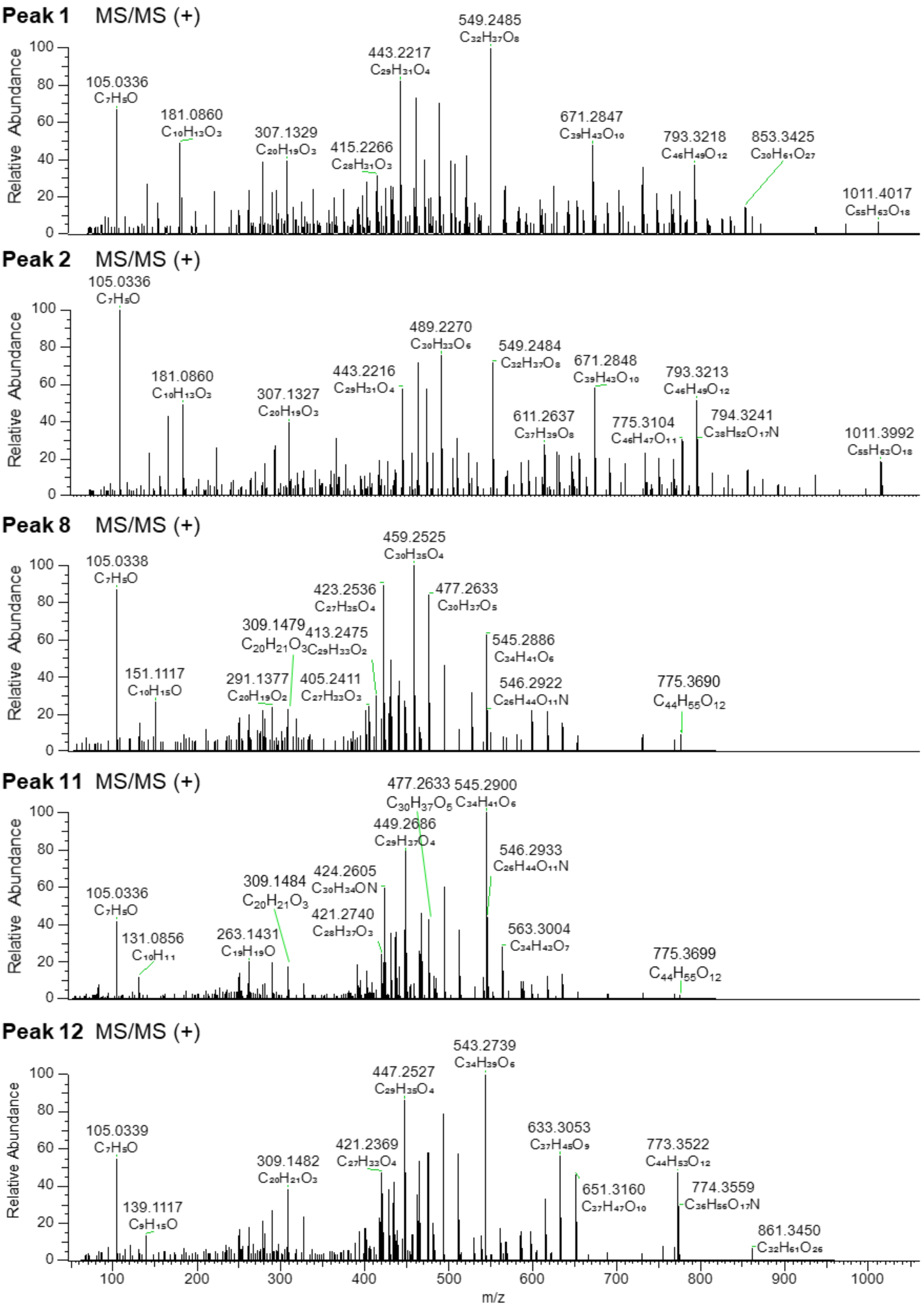

Figure S26. Product ion spectra of peaks **1**, **2**, **8**, and **11** obtained from the protonated molecular ion peak and **12** in obtained from the ammonium adduct ion peak positive ion mode.

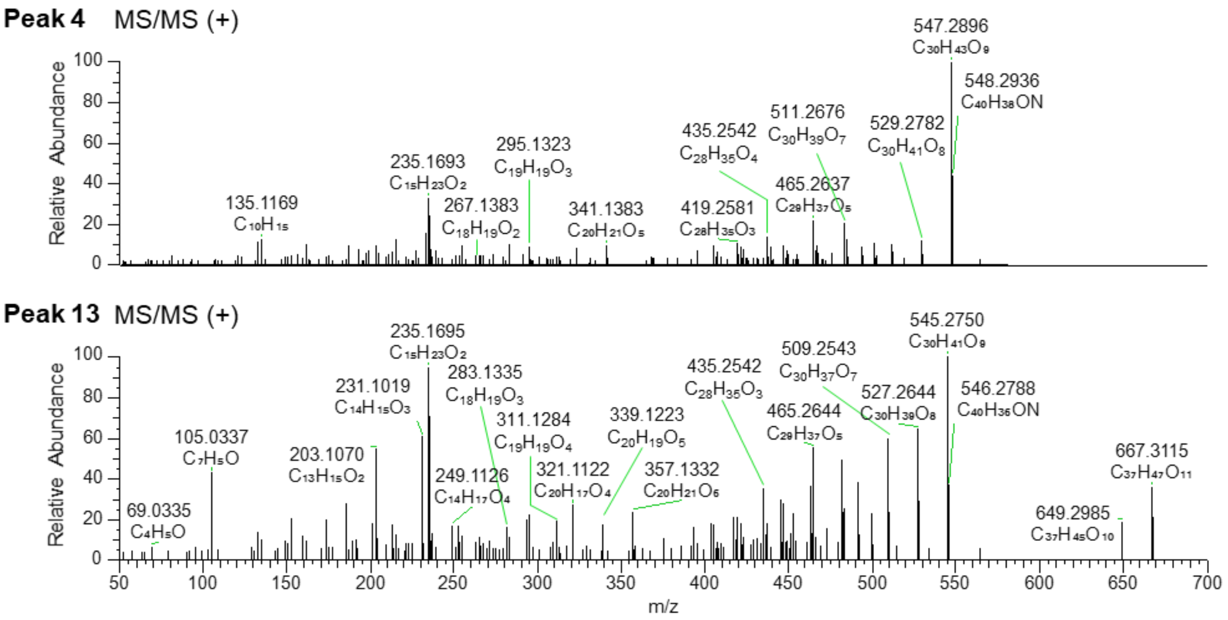

Figure S27. Product ion spectra of peaks **4** and **13** obtained from the protonated molecular ion peak.
